# Transcriptome Analysis of Gene Expression in *Dermacoccus abyssi* HZAU 226 under Lysozyme Stress

**DOI:** 10.3390/microorganisms8050707

**Published:** 2020-05-11

**Authors:** Xinshuai Zhang, Yao Ruan, Wukang Liu, Qian Chen, Lihong Gu, Ailing Guo

**Affiliations:** 1 College of Food Science and Technology, Huazhong Agricultural University, Wuhan 430000, China; hnzhangxinshuai@163.com (X.Z.); ruanyao12@webmail.hzau.edu.cn (Y.R.); liu_wukang@126.com (W.L.); qianchenh@163.com (Q.C.); lihongguu@163.com (L.G.); 2 National Research and Development Center for Egg Processing, Wuhan 430000, China

**Keywords:** *Dermacoccus abyssi* HZAU 226, RNA-seq, lysozyme treatment, tolerance mechanism

## Abstract

Lysozyme acts as a kind of cationic antimicrobial protein and effectively hydrolyzes bacterial peptidoglycan to have a bactericidal effect, which also plays an important role in protecting eggs from microbial contamination. *Dermacoccus abyssi* HZAU 226, a Gram-positive bacterium isolated from spoiled eggs, has egg white and lysozyme tolerance, but its survival mechanism is unknown, especially from a transcriptomics point of view. In this study, the high lysozyme tolerance of *D. abyssi* HZAU 226 was characterized by three independent experiments, and then the Illumina RNA-seq was used to compare the transcriptional profiles of this strain in Luria–Bertani (LB) medium with and without 5 mg/mL lysozyme to identify differentially expressed genes (DEGs); 1024 DEGs were identified by expression analysis, including 544 up-regulated genes and 480 down-regulated genes in response to lysozyme treatment. The functional annotation analysis results of DEGs showed that these genes were mainly involved in glutathione biosynthesis and metabolism, ion transport, energy metabolism pathways, and peptidoglycan biosynthesis. This study is the first report of bacterial-related lysozyme RNA-seq, and our results help in understanding the lysozyme-tolerance mechanism of bacteria from a new perspective and provide transcriptome resources for subsequent research in related fields.

## 1. Introduction

The taxonomy of *Dermacoccus abyssi* in the LPSN (List of Prokaryotic names with Standing in Nomenclature) database was as follows: Bacteria, Actinobacteria, Micrococcales, *Dermacoccaceae*, *Dermacoccus*, and the type strain was MT1.1 (= DSM 17573, = NCIMB 14084) [1]. Wasu et al. [2] first isolated *D. abyssi* from Mariana Trench deep water sediments at a depth of 10,898 m, which was a novel species in the genus *Dermacoccus*. Subsequent studies on the secondary metabolites and the biodecolorization of a food azo dye about this strain were reported [3,4]. Recently, two strains of *D. abyssi* were successfully isolated from silver carp fillets in the early storage period [5]. *D. abyssi* HZAU 226, which was used in this study, was isolated from spoiled eggs by traditional microbial culture method in our laboratory, and it had a strong tolerance to lysozyme [6].

Lysozyme is widely found in organisms and plays an important role in the natural immune system. The bactericidal activity can be attributed to its ability to effectively hydrolyze the peptidoglycan (PG) of bacterial cell walls, and its hydrolysis site is a β-1,4 glycosidic bond between *N*-acetylmuramic acid (NAM) and *N*-acetylglucosamine (NAG) [7]. In addition, lysozyme, as a kind of cationic antibacterial protein, can perforate the negatively charged bacterial cell membrane to form regular ion channels and destroy the bacterial cell membrane structure, causing the cell contents to flow out and the death of bacteria [8,9,10]. At present, in the food industry, lysozyme is mainly used for the preservation of various foods, and its effectiveness had been widely proved [11].

Given the widespread distribution and effective antibacterial activity of lysozyme, it was not surprising that some microorganisms, mainly pathogenic bacteria, had evolved a wide range of mechanisms to escape sterilization. There had been some reports on the research and review of lysozyme-resistance mechanisms, mainly including peptidoglycan modification and lysozyme inhibitor [12,13]. Peptidoglycan (PG), the major component of the cell wall, is essential for bacterial survival. Its proper modification can improve lysozyme tolerance [14]. *N*-deacetylation refers to the deacetylation of the C-2 residue of *N*-acetylmuramic acid (NAM) or *N*-acetylglucosamine (NAG) under the action of enzymes, a variety of *N*-deacetylases including PgdA, PdaV and PdaC had been identified in diverse bacteria [15,16]. Similarly, *O*-acetylation of PG refers to the addition of acetyl group to the C-6 hydroxyl group of NAM in most bacteria, which prevents the binding of lysozyme and peptidoglycan through steric hindrance [16,17]. In addition, teichoic acid modification and glutamic amidation in the peptide chain can also enhance lysozyme resistance [18,19]. Lysozyme inhibitor is another strategy that blocks the active site by binding to the lysozyme [20,21,22]. Transcriptional regulators in bacteria regulate the expression level of resistance factor genes to better survive in adverse environments such as lysozyme [23,24,25]. The study of the lysozyme-resistance mechanism has a positive effect on the development of more effective and comprehensive food preservatives, the development of antibacterial drugs and clinical treatment [26,27].

Currently, gene knockout and liquid chromatography–mass spectrometry (LC–MS) are the primary means of research in this field, but only one or a few genes can be studied in lysozyme tolerance at a time, and there is no systematic and comprehensive study to reveal the possible response mechanism of microorganisms to lysozyme stress. In the past decade, RNA-seq had become an important tool for analyzing differentially expressed genes (DEGs) in the whole transcriptome, which could comprehensively and quickly obtain almost all transcriptional sequence information of a species in a certain state [28]. Based on these, RNA-seq has been widely used in the study of tolerance mechanisms of microorganisms [29,30]. The whole genome sequence of *D. abyssi* HZAU 226 has been obtained by Nanopore sequencing technology and uploaded to National Center for Biotechnology Information (NCBI) under the accession numbers CP043031 and CP043032. In this study, we applied the Illumina RNA-seq technology to reveal the gene expression changes in Luria–Bertani (LB) medium (with added lysozyme), and performed functional analysis of DEGs, revealing the main response mechanism based on enrichment results. This study innovatively elaborated the lysozyme response mechanism from the genomic level, and also provided a basis for subsequent research in related fields.

## 2. Materials and Methods 

### 2.1. Characterization of Lysozyme Resistance 

In this study, three independent experiments characterized the high lysozyme tolerance of *D. abyssi* HZAU 226. *Staphylococcus aureus* ATCC 27217 was used as the control strain. Both strains were kept in a laboratory freezer at −80 °C. The experiments were performed in two parallel and three replications.

**Survival testing**. *D. abyssi* and *S. aureus* were inoculated in LB medium (Hope Bio-Technology, Qingdao, China) at 37 °C for 14 h, which were used to inoculate 20 mL LB medium with and without 5 mg/mL lysozyme (1% *v/v*), controlling the concentration of bacterial solution to 2–4 × 10^5^ colony-forming units (CFU)/mL. Then strains were grown at 37 °C with 50 RPM, 100 μL were plated on plate count agar (PCA) plates (Hope Bio-Technology) at 3 h, 6 h, and 9 h, incubated at 37 ℃ for 24 h before plate counting. Lysozyme was purchased from Sinopharm Chemical Reagent Co., Ltd (Shanghai, China).

**Inhibition zone testing**. We poured 5 mL Muellle-Hinton (MH) Agar medium (Hope Bio-Technology) into the plates, after solidification, put Oxford cups at an equal distance, and then poured 20 mL MH Agar medium inoculated overnight cultures (1% *v/v*) into the plates. After solidification, we removed the Oxford cups, added 100 μL lysozyme solutions at different concentrations to each hole, and added sterile saline to the control hole. The plates were allowed to stand for 30 min and incubated at 37 °C for 18–24 h.

**Scanning Electron Microscope (SEM) analysis**. *D. abyssi* and *S. aureus* were inoculated in LB medium at 37 °C, which were harvested at the logarithmic phase by centrifugation at 4 °C at 4000 RPM for 10 min and washed twice with phosphate buffer saline (PBS, pH 7.4). The experimental group was added with 0.2 mg/mL lysozyme solution and shaken for 3 h at 37 °C; the control group was added with an equal volume of saline. The concentration of the bacterial solution was controlled at 10^7^–10^8^ CFU/mL. After the treatment, centrifuged at 4000 RPM for 15 min, the supernatant was removed, and the bacteria enriched into 1.5 mL centrifuge tubes, which were quickly placed in 2.5% glutaraldehyde solution and fixed at 4 °C overnight, After centrifugation at 4000 RPM for 10 min, the supernatant was removed, and the cells were washed three times with PBS, which were then sequentially dehydrated in 30%, 50%, 70%, and 100% ethanol. Finally, the cells were dissolved in 200 μL tert-Butanol and freeze-dried, and the dried powder samples were observed for morphology by scanning electron microscope (JSM-6390LV, JEOL, Tokyo, Japan).

### 2.2. Lysozyme Treatment, RNA Extraction, Illumina Library Construction and Sequencing

*D. abyss* HZAU 266 was inoculated in LB medium at 37 °C for 14 h, which were used to inoculate 20 mL LB medium with (experimental group) and without (control group) 5 mg/mL lysozyme (1% *v/v*). Bacteria were harvested at the logarithmic phase by centrifugation at 4 °C at 4000 RPM for 10 min and washed twice with PBS. Both the experimental group and the control group performed three biological replicates.

Total RNA of each sample was extracted using TRIzol Reagent (Invitrogen, Carlsbad, CA, USA)/RNeasy MiniKit (Qiagen, Hilden, Germany). Total RNA was quantified and qualified by Agilent 2100 Bioanalyzer (Agilent Technologies, Palo Alto, CA, USA), NanoDrop (Thermo Fisher Scientific Inc., Waltham, MA, USA) and 1% agarose gel. 1μg total RNA with RNA integrity number (RIN) value above 6.5 was used for following library preparation. Next generation sequencing library preparations were constructed according to the manufacturer’s protocol. The rRNA was depleted from total RNA using Ribo-Zero rRNA removal kit (Bacteria, Illumina, San Diego, CA, USA). The ribosomal depleted RNA was then fragmented and reverse-transcribed. First strand cDNA was synthesized using ProtoScript II Reverse Transcriptase with random primers and actinomycin D. The second-strand cDNA was synthesized using Second Strand Synthesis Enzyme Mix (include dACG-TP/dUTP). The purified double-stranded cDNA by beads was then treated with end prep enzyme mix to repair both ends and add a dA-tailing in one reaction, followed by a T-A ligation to add adaptors to both ends. Size selection of adaptor-ligated DNA was then performed using beads, and fragments of ~420 bp (with the approximate insert size of 300 bp) were recovered. The dUTP-marked second strand was digested with Uracil-Specific Excision Reagent enzyme. Each sample was then amplified by polymerase chain reaction (PCR) for 13 cycles using P5 and P7 primers, with both primers carrying sequences which can anneal with flow cell to perform bridge PCR and P7 primer carrying a six-base index allowing for multiplexing. The PCR products were cleaned up using beads, validated using an Qsep100 (Bioptic, New Taipei City, Taiwan), and quantified by Qubit3.0 Fluorometer (Invitrogen, Carlsbad, CA, USA). 

Libraries with different indices were then multiplexed and loaded on an Illumina HiSeq instrument according to the manufacturer’s instructions (Illumina, San Diego, CA, USA). Sequencing was carried out using a 2 × 150 paired-end (PE) configuration; image analysis and base calling were conducted by the HiSeq Control Software (HCS) + OLB + GAPipeline-1.6 (Illumina) on the HiSeq instrument. The sequences were processed and analyzed by GENEWIZ. All transcriptome raw data has been deposited at the NCBI Sequence Read Archive (SRA) database under the accession PRJNA600111.

### 2.3. Sequence Analysis and Functional Annotation

Quality control of raw data was processed by Cutadapt (version 1.9.1). Firstly, reference genome sequences and gene model annotation files of relative species were downloaded from NCBI. Secondly, Bowtie2 (v2.2.6) was used to index the reference genome sequence. Finally, clean data were aligned to reference genome via software Bowtie2. In the beginning, transcripts in fasta format were converted from known gff annotation file and indexed properly. Then, with the file as a reference gene file, HTSeq (v0.6.1p1) estimated gene expression levels from the pair-end clean data. Differential expression analysis used the DESeq2Bioconductor package, a model based on the negative binomial distribution. After being adjusted by Benjamini and Hochberg’s approach for controlling the false discovery rate, the adjusted *p*-value (padj) of genes was set at <0.05 to detect differentially expressed ones. Rockhopper uses a Bayesian approach to create a transcriptome map including transcription start/stop sites for protein coding genes and novel transcripts identified by Rockhopper. Blast intergenic novel transcripts to the Non-Redundant Protein Sequence (NR) database, non-annotated transcripts are considered as potential trans-encoded sRNAs. A novel antisense transcript was treated as cis-encoded sRNA. 

GOSeq (v1.34.1) was used to identify Gene Ontology (GO) terms that annotate a list of enriched genes with a significant *p*-value less than 0.05. We used topGO to plot DAG. Blastp (Version 2.7.1+) and rpsblast (Version 2.7.1+) were used to compare the coding protein to the Cluster of Orthologous Groups of proteins (COG) database (cut off e-value ≤ 1 × 10^−5^). This study used scripts in house to enrich significant DEGs in Kyoto Encyclopedia of Genes and Genomes (KEGG) pathways.

## 3. Results

### 3.1. High Lysozyme Resistance of *D. abyssi* HZAU 226

**Figure 1:**
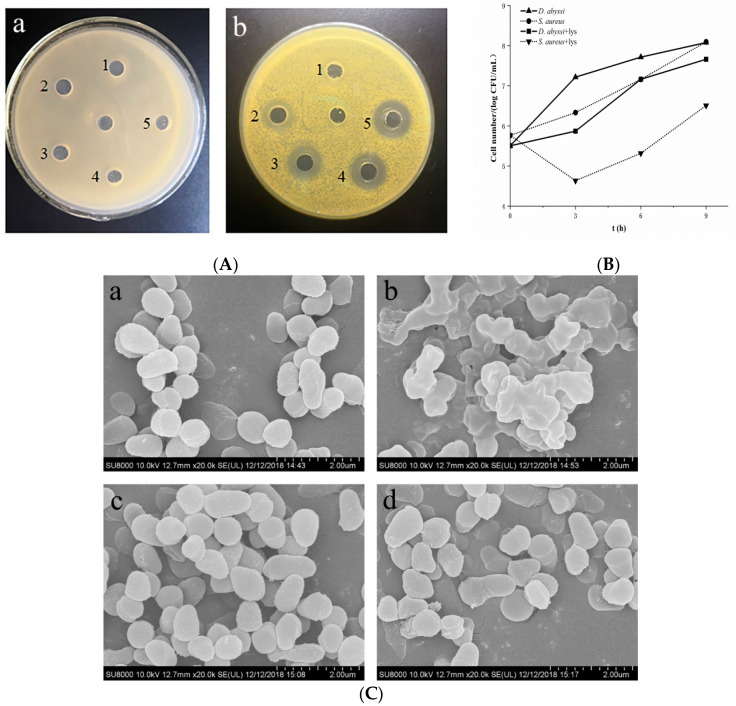
(**A**) The survival curve of *D. abyssi* HZAU 226 and *S. aureus* ATCC 27,217 in Luria–Bertani (LB) medium with and without 5 mg/mL lysozyme. Data were obtained from two independent experiments using triplicate samples per each experiment via plate counts at different time points; (**B**) a and b were the lysozyme inhibition zone test of *D. abyssi* HZAU 226 and *S. aureus* ATCC 27217. The concentration of lysozyme in pores 1–5 were 1.5 mg/mL, 3.5 mg/mL, 5.5 mg/mL, 7.5 mg/mL and 10 mg/mL respectively, and central pore was saline control; (**C**) Scanning electron microscope (SEM) images of lysozyme treatment. Note: control group: a (*S. aureus*) and c (*D. abyssi*); experimental group: b (*S. aureus*) and d (*D. abyssi*).

The growth of *S. aureus* was significantly inhibited compared with the LB medium without lysozyme at 3 h, viable counts reduced to 4.6 log CFU/mL, and then gradually increased. Conversely, *D. abyssi*, even in the LB medium with lysozyme, had been growing positively, and viable counts were 5.87 log CFU/mL at 3 h, greater than the initial inoculum (5.5 log CFU/mL). However, compared with the 7.22 log CFU/mL of standard LB medium, the growth of *D. abyssi* was inhibited to a certain extent (Figure 1A).

The results of the inhibition zone testing showed that *D. abyssi* did not produce transparent circles on the plates with different concentrations of lysozyme, while the control strain *S. aureus* produced transparent circles of various sizes, and transparent circle diameter was proportional to the concentration (Figure 1B).

*D. abyssi* and *S. aureus* were treated with 0.2 mg/mL lysozyme for 3 h, the change of bacterial surface morphology was observed by scanning electron microscopy (SEM). The surface of two strains without lysozyme treatment were all smooth, full-bodied, and clear spheres or ellipsoids. After lysozyme treatment, the morphology of *S. aureus* cells changed greatly, the cell membrane/wall was twisted and sunken, the boundary between the cells was blurred, and the whole-cell morphology collapsed completely. However, for *D. abyssi*, only a few bacterial cells wall showed wrinkles, but still maintained complete cell morphology, and the structure was not significantly changed compared to the control group (Figure 1C). It was concluded that *D. abyssi* HZAU 226 had high lysozyme resistance from three independent lysozyme tolerance experiments.

### 3.2. Quality Control of Sequencing Data

After constructing 6 cDNA libraries and performing RNA-seq with the Illumina HiSeq platform, which yielded 296.10 million reads in total with a 2 × 150 PE configuration. After removing technical sequences, including adapters, PCR primers, or fragments thereof, and a quality of bases lower than 20, a total of 242.67 million clean reads were obtained, and Q30 of the base ratio were higher than 90.80%, indicating that the amount and quality of RNA-seq data were high, which provided for subsequent reference genome comparison analysis reliable data source. The ratio of reads mapping to the reference genome was high, with a mapping rate that fell into a range between 86.33% and 94.70% (Table 1).

### 3.3. Identified New Genes and sRNA by Transcriptome Analysis

RNA-seq technology is an effective way to identify new genes in the genome, annotations of transcripts in existing databases may not be comprehensive, new transcripts can usually be detected by RNA-seq technology [31]. In this study, 284 new transcripts were identified in the *D. abyssi* HZAU 226 genome, of which 271 were annotated as antisense transcripts. These new transcripts were distributed on chromosome and plasmid (Table S1).

The bacterial small regulatory RNA (sRNA) prediction was also performed on the sequencing data. sRNAs are a class of 40–400 nt non-coding RNAs. They do not encode proteins, which are important regulators of bacterial life activities and interact with mRNA or proteins to affect gene expression, including cis-encoded sRNA and trans-encoded sRNA [32]. In this study, a total of 252 potential sRNAs were identified, most of which were cis-encoded sRNAs, which were distributed in chromosome and plasmid (Table S2).

### 3.4. Differentially Expressed Genes’ (DEGs) Response to Lysozyme Treatment

**Figure 2:**
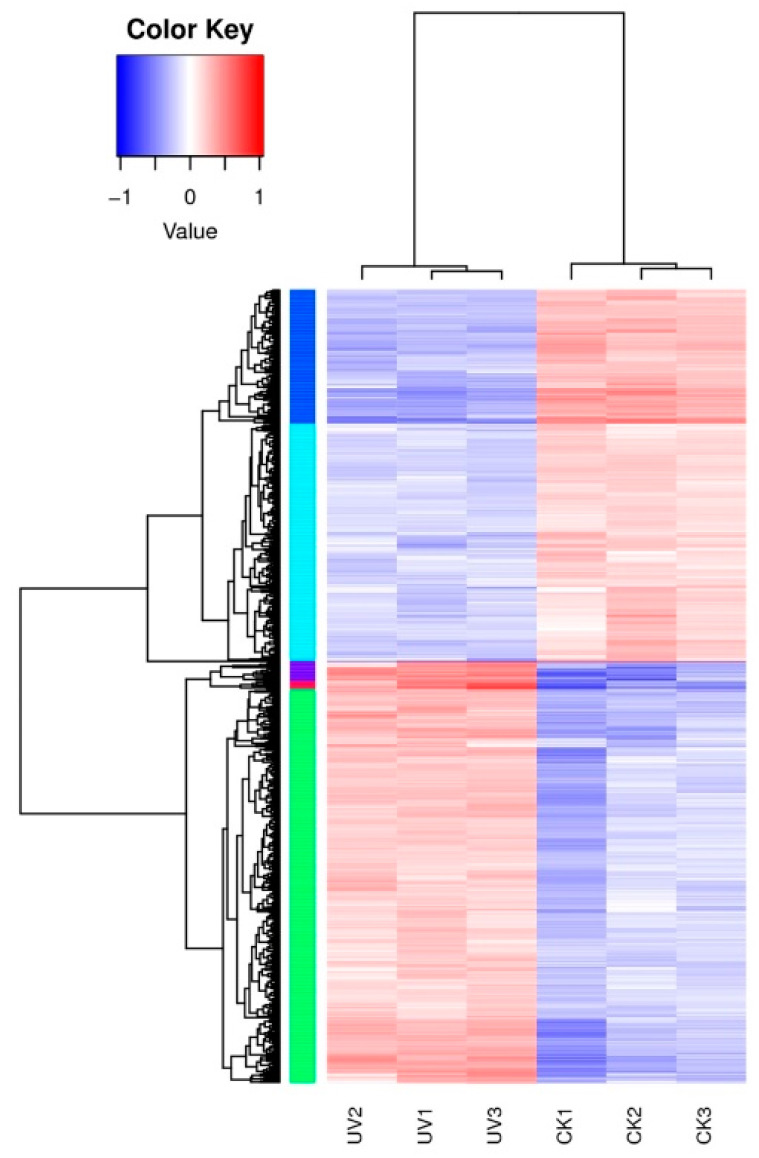
Heatmap diagrams showed the relative expression levels of total differentially expressed genes (DEGs) among the two treatments. Note: CK: LB medium without lysozyme; UV: LB medium with lysozyme.

To evaluate the relative level of gene expression in *D. abyssi* under control or lysozyme treatment, fragments per kilobase per million (FPKM) values were calculated based on the uniquely mapped reads. The FPKM values distributions of genes in six samples are shown in Figure S1, with the mean value of 399.05. Then, based on the negative binomial distribution by DESeq2 software [33], a part of the genes was identified as differentially expressed in lysozyme treatment samples: 544 genes were calculated as up-regulated and 480 filtered as down-regulated genes with the cutoff of padj < 0.05 and |log2(fold change)| > 1 (Table S3). Furthermore, a general overview of the expression pattern was visualized in a heat map (Figure 2), which provided an overall understanding of the changes in gene expression. The expression patterns in the control group and the experimental group were similar, both of which could be clustered together, indicating that their respective samples repeatability was good. In addition, the expression patterns of most DEGs in the CK and UV groups were completely opposite.

Table S4 listed top30 DEGs with the lowest adjusted *p*-value (padj), including 9 up-regulated genes and 21 down-regulated genes. The up-regulated genes mainly include transporters, putative proteins (NPGAP_02910, NPGAP_14815) and aspartate aminotransferase (NPGAP_00785). Three of them were iron ion transporters. The iron ion binding protein Efeo (NPGAP_04750) can transfer Fe^3+^ to the iron permease FTR1 (EfeU) (NPGAP_04745), which transports Fe^3+^ into the cell for absorption and utilization [34]. FepB (NPGAP_13120) can secrete complex Fe^3+^ iron carriers to the extracellular environment [35]. Iron ions are indispensable for the growth of organisms and cofactors for many proteins to perform their functions, which also participate in the redox system [36]. The other two were sugar and thiamine transporters. Down-regulated genes include a variety of enzymes, transcription regulators, and transporters that drive different functions.

### 3.5. Functional Analysis of DEGs

**Figure 3:**
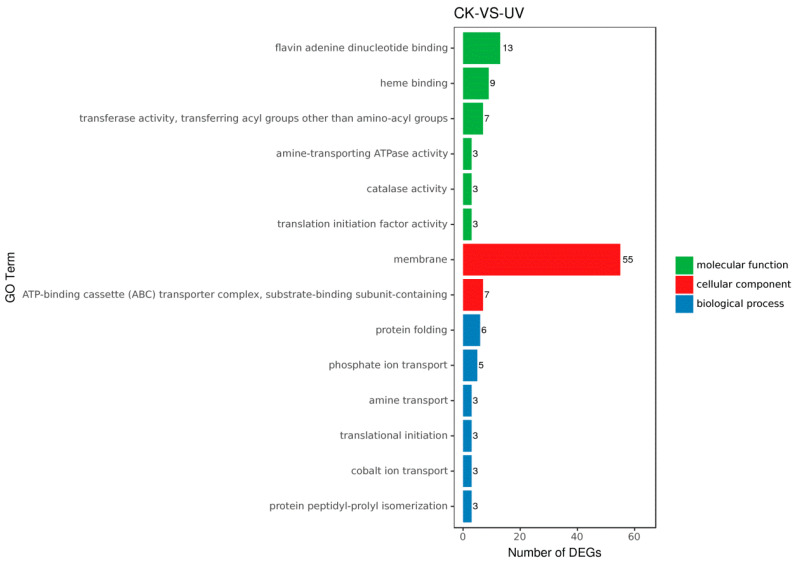
Gene Ontology (GO) analysis of DEGs in three main categories.

Gene Ontology (GO) is an internationally standardized gene function classification system. Significant enrichment analysis of GO functions by DEGs can better explore the physiological, metabolic functions and biological processes involved in DEGs. We annotated 548 out of 1024 DEGs to GO terms in the GO database, 309 up-DEGs and 239 down-DEGs. 14 GO terms significantly enriched 123 DEGs in response to lysozyme stress. Molecular function (MF) (GO: 0003674) enriched 38 DEGs, of which “flavin adenine dinucleotide binding”, “heme binding” and “catalase activity” terms indicate that lysozyme treatment was closely related to oxidative stress, and also included some transferases. Cellular component (CC) (GO: 0005575) enriched 62 DEGs, which were closely related to the transmembrane transport system including the ABC transporter. Biological process (BP) (GO: 0008150) enriched 23 DEGs, which were mainly used for ion transport and protein synthesis (Figure 3). The directed acyclic graph (DAG) of GO enrichment analysis of DEGs showed that BP was significantly enriched in “nucleoside phosphate biosynthetic process” (GO: 1901293) and “transition metal ion transport” (GO: 0000041) (Figure S2); Cellular component (CC) was significantly enriched to “ATP-binding cassette transporter complex” (GO: 0043190) (Figure S3); MF was eventually significantly enriched to “flavin adenine dinucleotide binding” (GO: 0050660), “ATPase activity, coupled to transmembrane movement of substances” (GO: 0042626) and “transition metal ion transmembrane transporter activity” (GO: 0046915) (Figure S4).

**Figure 4:**
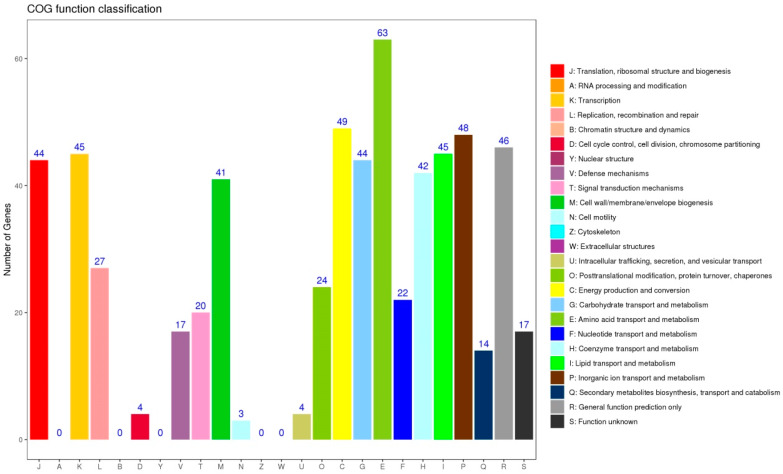
Cluster of Orthologous Groups of proteins (COG) analysis of DEGs between CK and lysozyme treatments.

The COG database is a genome-scale protein function and evolutionary analysis tool capable of homologous classification of gene products. COG annotation results (Figure 4) showed that 556 DEGs were assigned to 20 COG terms. The “Amino acid transport and metabolism” (11%) term was enriched with the most genes, and this indicated that under the conditions of lysozyme treatment, the rate of the strain to utilize and metabolise amino acids has changed, amino acids are generally used to synthesize proteins and generate energy during metabolism, and the DEGs of the “Energy production and conversion” term had also verified the result. In addition, multiple functional terms were enriched with more than 40 DEGs. The DEGs of “P” term indicated that lysozyme treatment had an effect on the cell’s ion metabolism and transport, which could be explained by lysozyme as a kind of cationic antibacterial protein capable of forming regular ion channels with negatively charged bacterial cell membranes, resulting in more frequent ion exchange [9,10], consistent with GO enrichment analysis results.

**Figure 5:**
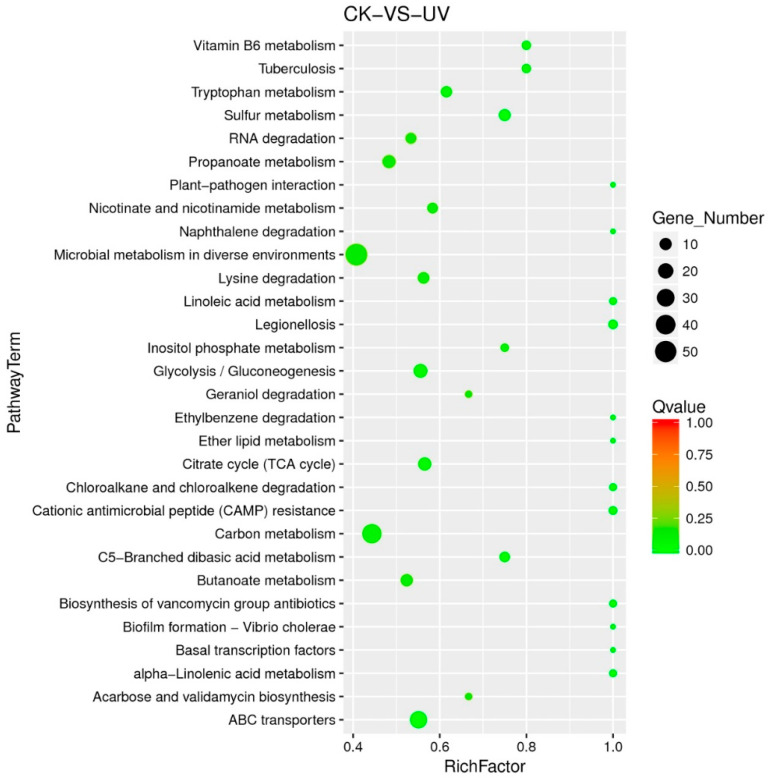
Scatter plot of Kyoto Encyclopedia of Genes and Genomes (KEGG) pathway enrichment for DEGs. Rich factor referred to the ratio of the number of genes annotated to the pathway in the DEGs to the total number of genes located at the pathway among all annotated genes.

Different genes in the organism coordinate their biological functions, and in order to better explore the biochemical metabolic pathways and signal transduction pathways involved in DEGs, KEGG pathway enrichment analysis was conducted; 257 DEGs were enriched into 109 different pathways, and 30 pathways with the most significant enrichment (the lowest Qvalue) were selected for display (Figure 5). “Carbon metabolism” enriched 39 DEGs, indicating that the carbon source utilization efficiency of the strain had changed, affecting the growth of the strain. “Ribosome” (6.37%) and “Aminoacyl-tRNA biosynthesis” (4.12%) enriched multiple up-regulated genes, indicating that lysozyme treatment stimulated the ribosome synthesis pathway and accelerated protein synthesis. Many DEGs were enriched by various amino acid metabolic pathways, which was consistent with the results of COG annotation. Five pathways of “Replication and repair” enriched multiple down-regulated genes, including the homologous recombination repair proteins RecF and RecO. In addition, the “ABC transporters” and “Quorum sensing” enriched with a variety of transporters, including the peptide transporter, the five-subunit CbiMNQO complex of nickel-cobalt transport across the membrane (GO: 0006824), and the sugar and branched chain amino acid transport systems GanOPQ and LivKHMGF complex, but no report has been published yet, and further analysis is needed to reveal their relationship with lysozyme stress.

## 4. Discussion

In this study, the high tolerance of *D. abyssi* HZAU 226 to lysozyme was successfully characterized by survival testing, inhibition zone testing and scanning electron microscope. In order to explore the genes involved in the lysozyme tolerance mechanism, we analyzed the Illumina mRNA-seq data from this strain grown in LB medium with the addition of 5 mg/mL lysozyme. Starting from 6 sequenced RNA libraries, we identified a potential 284 new transcripts, 252 sRNAs, and 1024 DEGs. The functional analysis of GO, COG and KEGG enrichment could better understand the function and response mechanism of DEGs. These genes were mainly involved in the biosynthesis and metabolism of glutathione, ion transport, energy metabolism pathways and peptidoglycan biosynthesis, which had a strong response to lysozyme treatment.

**Figure 6:**
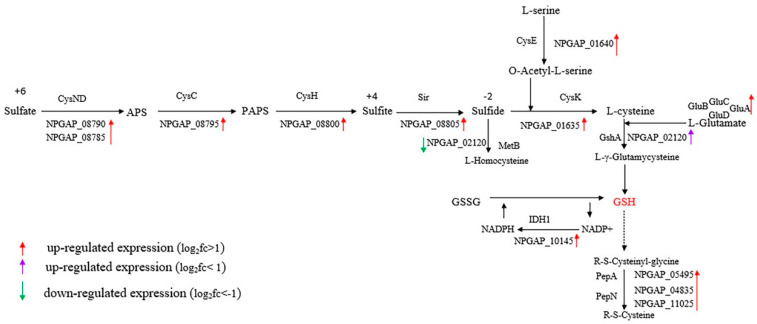
Glutathione biosynthesis and metabolism.

The first subgroup of lysozyme-responsive genes was glutathione biosynthesis and metabolism. Glutathione is a tripeptide consisting of glutamic acid, cysteine, and glycine. The thiol group on cysteine is a glutathione active group, which has the function of anti-free radical and anti-oxidative stress, and plays a key role in the regulation of redox signal transduction. Glutathione comes in two forms: reduced (GSH) and oxidized (GSSG), with a GSH/GSSG ratio of about 100/1 [37,38]. After lysozyme treatment, genes, related to glutathione biosynthesis and metabolism, were significantly up-regulated. The first was that the synthesis rate of L-cysteine, one of the raw materials, was accelerated, and the four reductase-encoding genes (NPGAP_08785, NPGAP_08790, NPGAP_08795, NPGAP_08800, NPGAP_08805) in the assimilation sulfate reduction pathway were all overexpressed, cysteine synthetase (*cysK*, NPGAP_01635: log2FoldChange (L_2_fc) = 2.19) was also significantly up-regulated, catalyzing H_2_S to produce L-cysteine. The rate of H_2_S-generated L-homocysteine slowed down. The serine O-acetyltransferase (*cysE*, NPGAP_01640: L_2_fc = 2.12) catalyzed the production of cysteine by serine (Figure 6).

In the glutathione metabolism pathway, the expression of the isocitrate dehydrogenase gene (NPGAP_10145: L_2_fc = 2.13) was up-regulated more than four times, which catalyzed the reduction of NADP^+^ to NADPH, speeding up the hydrogen supply to GSSG and increasing the rate of GSH synthesis [39]. *D. abyssi* had the complete Pentose phosphate pathway, but the expression of the 6-phosphogluconate dehydrogenase gene (NPGAP_14270: L_2_fc = 0.31) in this pathway changed little, which was a catalytic enzyme producing a NADPH reaction. The glutamate-cysteine ligase gene (NPGAP_02120: L_2_fc = 0.78), which catalyzes the production of GSH precursor L-γ-glutamycysteine, was up-regulated almost twice. The aminopeptidase genes (NPGAP_11025, NPGAP_04835, NPGAP_05495) were also significantly up-regulated, which cleaved the protease after S-H of GSH was activated, indicating increased metabolism of GSH (Figure 6). The DEGs’ enrichment results of the ABC transporters pathway were consistent with the above conclusions. The expression of the gluABCD operator (NPGAP_08370, NPGAP_08375, NPGAP_08380, NPGAP_08385) responsible for glutamate transport was significantly up-regulated, indicating that the transport of glutamate, one of the raw materials of GSH, was accelerated. Thiamine transporter genes (NPGAP_02130, NPGAP_02135) were overexpressed, and thiamine pyrophosphate was an important cofactor for NADP^+^, NADPH, and GSH [40]. Another type of antioxidant enzyme catalase (GO: 0004096) was also significantly up-regulated (Figure 3). Therefore, a clear conclusion could be drawn: lysozyme treatment activated the organism’s oxidative stress, which accelerated the biosynthesis and metabolism of glutathione.

The second major class of lysozyme-responsive genes was ion transport. COG annotation results showed that ion transport and metabolism (“P” category) enriched 46 DEGs. The enrichment results of a two-component system (ko02020) pathway showed that the coding genes of the K^+^ transport Kdp system were down-regulated to varying degrees, among which the KdpA gene (NPGAP_12520: L_2_fc = −1.16) responsible for K^+^ transport and the ATPase KdpB gene (NPGAP_12515: L_2_fc = −1.00) providing energy for the transport process were significantly down-regulated, the response factor KdpE gene (NPGAP_12495: L_2_fc = −0.44) and the stabilizing protein KdpC gene (NPGAP_12510: L_2_fc = −0.84) were down-regulated less than two times, which we suspected was due to the cationic properties of lysozyme caused osmotic stress. The significant up-regulation of the OpuA gene (NPGAP_13435) and OpuBD genes (NPGAP_13430, NPGAP_13440) in the osmoprotectant transport system also validated this conclusion. In the hypertonic environment, bacteria balance osmotic pressure by self-synthesis or environmental absorption of compatible solute [41,42]. The phosphate specific transport (Pst) system (GO: 0006817) consists of PatS, PatC, PatA, PatB and PhoU, belonging to the ABC transporter family. Except for the ATP-binding protein PatB, the coding genes of the other four proteins were significantly up-regulated, and inorganic phosphate plays an important role in energy metabolism and intracellular signaling [43]. In addition, the iron transporter genes were significantly changed, and 7 of the 8 DEGs related to iron transport were significantly up-regulated (Table S5), including the trivalent iron transporter FepBDGC. Iron ions are widely involved in redox reactions in vivo as cofactors in protein-driving functions, most of which exist in the host hemepexin in the form of heme (GO: 0020037) [36]. H_2_O_2_ treatment of *Enterococcus faecalis*, *Streptococcus thermophilus* under heat shock, and lysozyme treatment of *Streptococcus gallolyticus* subsp. *gallolyticus* also induced the overexpression of such genes [44,45,46], which indicated that lysozyme treatment was related to the production of reactive oxygen species (ROS).

**Figure 7:**
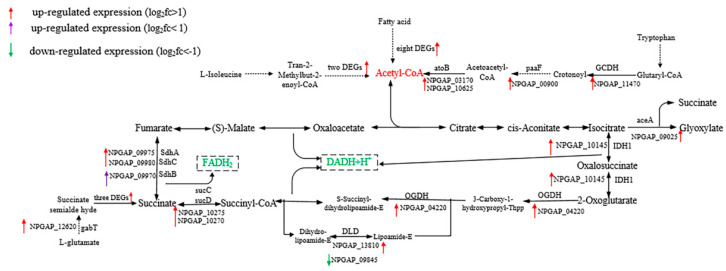
Energy metabolism pathway.

The third subgroup of lysozyme-responsive genes was the energy metabolism pathway. According to the KEGG enrichment pathway of DEGs, the citrate (TCA) cycle (ko00020) was significantly enhanced, and enriched with 6 up-regulated DEGs, including two key enzymes in TCA cycle: isocitrate dehydrogenase (NPGAP_10145: L_2_fc = 2.13) and 2-oxoglutarate dehydrogenase (NPGAP_04220: L_2_fc = 1.28), and the expression of another key enzyme, citate synthase (NPGAP_03845: L_2_fc = 0.43), was also up-regulated, but less than two times. Multiple genes involved in the biosynthesis of acetyl-CoA in fatty acid degradation (ko00071), tryptophan metabolism (ko00380) and valine, leucine and isoleucine degradation (ko00280) pathways were significantly up-regulated (Figure 7). Oxidative phosphorylation (ko00190), the main energy producing pathway of the organism, was also significantly enhanced (Figure S5). Multiple cytochrome enzyme and ATP synthetase genes were significantly up-regulated, but the NADH dehydrogenase gene was down-regulated by more than two times, possibly the H^+^ content increased due to the strengthening of TCA cycle. The isocitrate lyase gene (NPGAP_09025: L_2_fc = 2.72), a key rate-limiting enzyme in the glyoxylate cycle, was up-regulated more than six times. Studies had shown that under the pressure of survival of microorganisms, the glyoxylate cycle could start to utilize the carbon source in the environment to obtain energy and synthesize sugars, amino acids, nucleic acids and other biological macromolecules for survival [47,48]. The enhancement of the energy metabolism pathway indicated that lysozyme treatment increased the energy demand of the strain, and the synthesis of important products such as ATP, NADH and FADH_2_ was accelerated to provide more energy and coenzymes for biochemical reactions such as oxidative stress, which was consistent with the results of several RNA-seq studies related to tolerance [29,49,50].

The action site of lysozyme is the β-1,4-glycosidic bond on bacterial peptidoglycan. Therefore, peptidoglycan synthesis was defined as the fourth major class of response genes. Peptidoglycan biosynthesis (ko00550) enriched five DEGs, including *murB* (NPGAP_03210: L_2_fc = −1.60), *bcrC* (NPGAP_04140), *murE* (NPGAP_07060: L_2_fc = −1.16, NPGAP_00880: L_2_fc = 1.84) and *dacB* (NPGAP_12250). When studying the effect of *gpsB* on lysozyme resistance, Rismondo et al. [51] found that the rate of incorporation of cell wall precursors into the peptidoglycan network would affect the lysozyme resistance, and any mutation that reduced the production of peptidoglycan would increase *N*-deacetylation rate, thereby improving resistance to lysozyme. Therefore, the expression levels of Mur ligase in the peptidoglycan biosynthesis pathway were analyzed, the expressions of *murA* (L_2_fc = −0.94), *murB* and *murC* (L_2_fc = −0.79) were all down-regulated, while the expressions of *murD* and *murG* were basically unchanged, and the expression of *murF* (L_2_fc = 0.70) was up-regulated, suggesting that lysozyme limited the biosynthesis of bacterial peptidoglycan to some extent, which was consistent with the results of survival testing. Unfortunately, the peptidoglycan *N*-acetylglucosamine deacetylase gene (NPGAP_11765: L_2_fc = 0.23) in the genome had not changed significantly, the survival strategy of this strain needed further study. In addition, MurT and GatD are two enzymes responsible for amidation of peptidoglycan. The catalytic mechanism, crystal structure, and the effect of *murTgatD* operon expression on growth rate, β-lactam and lysozyme resistance have been reported [19,52,53]. In this study, the expressions of *murT* (L_2_fc = −0.95) and *gatD* (log2fc = −0.67) were both down-regulated, which reduced the degree of cross-linking of peptidoglycan. It is worth mentioning that although *D. abyssi* is Gram-positive bacteria, it has a complete DAP-peptidoglycan biosynthesis pathway, which does not require pentapeptide bridge connection. D-Ala-D-Ala carboxypeptidase gene (NPGAP_12250: L_2_fc = 1.61) in DAP-type peptidoglycan synthesis pathway was significantly up-regulated, resulting in a low cross-linking degree of peptidoglycan and loose reticular structure compared with other Gram-positive bacteria, which could reduce the sensitivity of lysozyme to a certain extent [54,55].

## 5. Conclusions

In summary, the high lysozyme tolerance of *D. abyssi* HZAU 226 was successfully characterized by lysozyme sensitivity experiments. To explore the specific adaptation mechanism to lysozyme, we used high-throughput sequencing technology to perform transcriptome analysis of *D. abyssi* HZAU 226 under lysozyme treatment, which is also the first report of microbial-related lysozyme RNA-seq technology; 1024 DEGs were screened and enriched with the GO, COG and KEGG databases. The results provided the basis for the subsequent verification of specific protein functions, and provided valuable information for the survival of this strain in eggs and subsequent lysozyme tolerance analysis of other microorganisms.

## Figures and Tables

**Table 1:** Data quality statistics and reference sequence matching after filtering.

Sample	Sample Description	Total Reads	Bases	Q20 ^1^ (%)	Q30 ^2^ (%)	GC ^3^ (%)	Total Mapped
CK1	Control replication 1	33,366,114	4,934,245,447	96.80	91.36	67.43	31,528,403 (94.4923%)
CK2	Control replication 2	33,483,220	4,948,473,732	96.61	90.91	67.29	31,529,020 (94.1636%)
CK3	Control replication 3	34,522,436	5,128,803,026	97.78	93.92	67.40	32,692,711 (94.6999%)
UV1	Lysozyme treatment replication 1	56,645,366	8,421,131,651	98.17	94.79	66.31	51,680,501 (91.2352%)
UV2	Lysozyme treatment replication 2	45,945,296	6,769,856,909	96.53	90.80	64.46	39,814,219 (86.6557%)
UV3	Lysozyme treatment replication 3	38,707,694	5,712,404,289	96.60	90.93	64.77	33,415,978 (86.3290%)

^1^ Percentage of bases with a Phred value of at least 20. ^2^ Percentage of bases with a Phred value of at least 30. ^3^ Total number of bases G and C as a percentage of total number of bases.

## Supplementary Materials

The following are available online at https://www.mdpi.com/2076-2607/8/5/707/s1. Figure S1: The FPKM density distribution, Figure S2: Directed Acyclic Graph of GO (BP) enrichment analysis, Figure S3: Directed Acyclic Graph of GO (CC) enrichment analysis, Figure S4: Directed Acyclic Graph of GO (MF) enrichment analysis, Figure S5: The KEGG pathways (ko00190) “Oxidative phosphorylation” mapped with DEGs, Table S1: New transcript prediction results, Table S2: sRNA structure annotation results, Table S3: 1024 DEGs and functional annotations, Table S4: Top30 DEGs with the lowest adjust *p*-value (padj) under lysozyme treatment, Table S5: The DEGs involved in iron transporter.
