# Supplementary material for: Transcriptome Analysis of Gene Expression in Dermacoccus abyssi HZAU 226 under Lysozyme Stress
Source: Microorganisms. 2020 May 11;8(5):707. doi: 10.3390/microorganisms8050707 (PMC7286019; doi:10.3390/microorganisms8050707)
Supplement: Supplementary file 1 [file microorganisms-08-00707-s001.zip › Supplementary_Figure S1-S5.pdf]

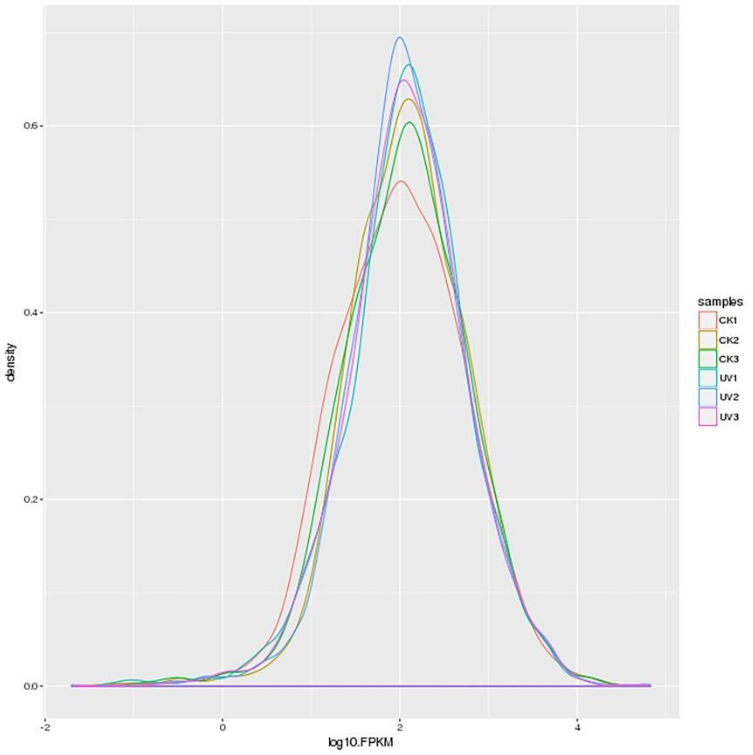

Figure S1. The FPKM density distribution. The abscissa was log10 (FPKM), the higher the value, the higher the gene expression level. The ordinate was the density of genes, which was equal to the number of genes corresponding to the amount of expression on the abscissa. Different colors represented different samples. The peak of the curve represented the region where the gene expression was most concentrated.

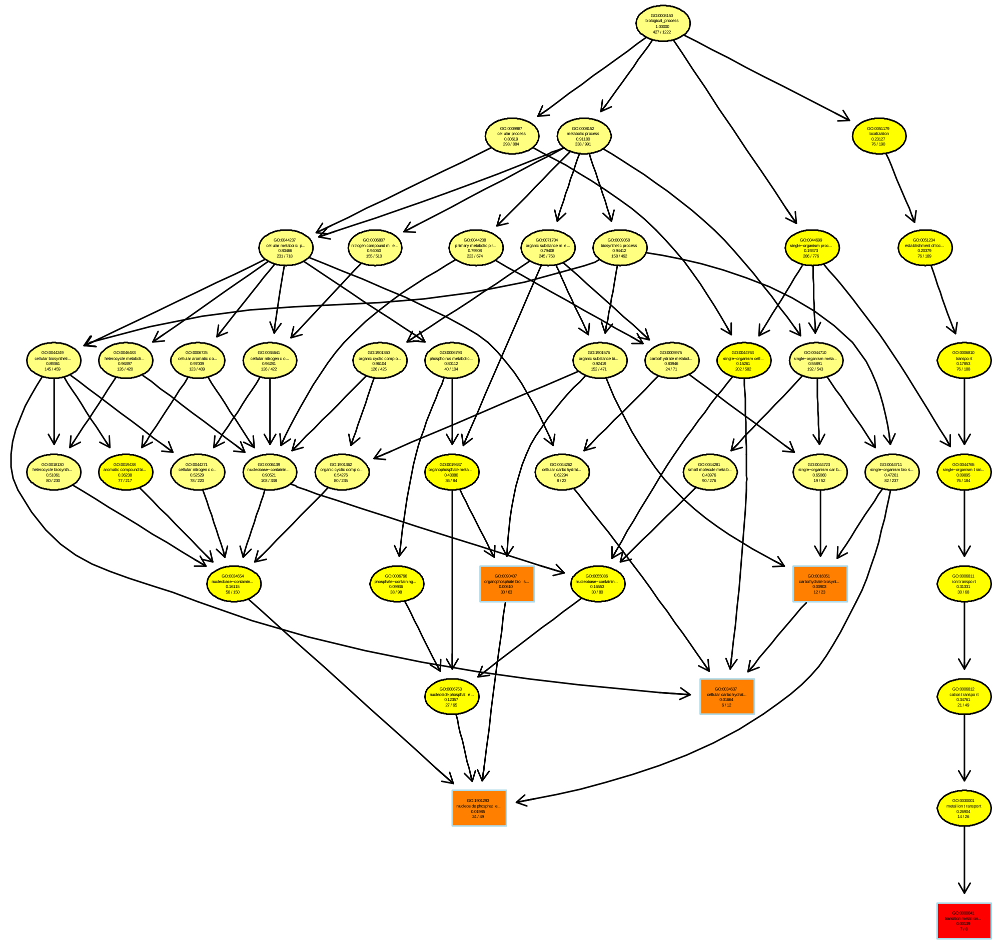

Figure S2. Directed Acyclic Graph of GO (BP) enrichment analysis.

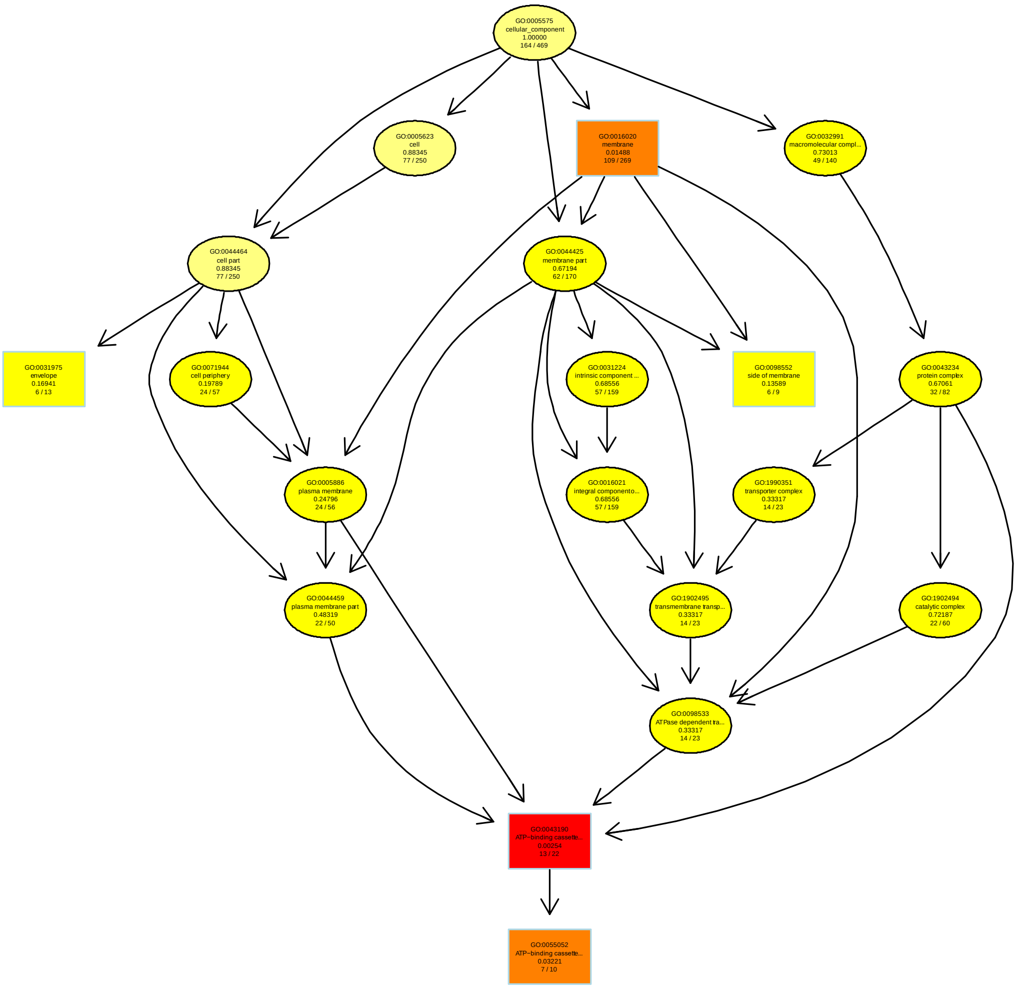

Figure S3. Directed Acyclic Graph of GO (CC) enrichment analysis.

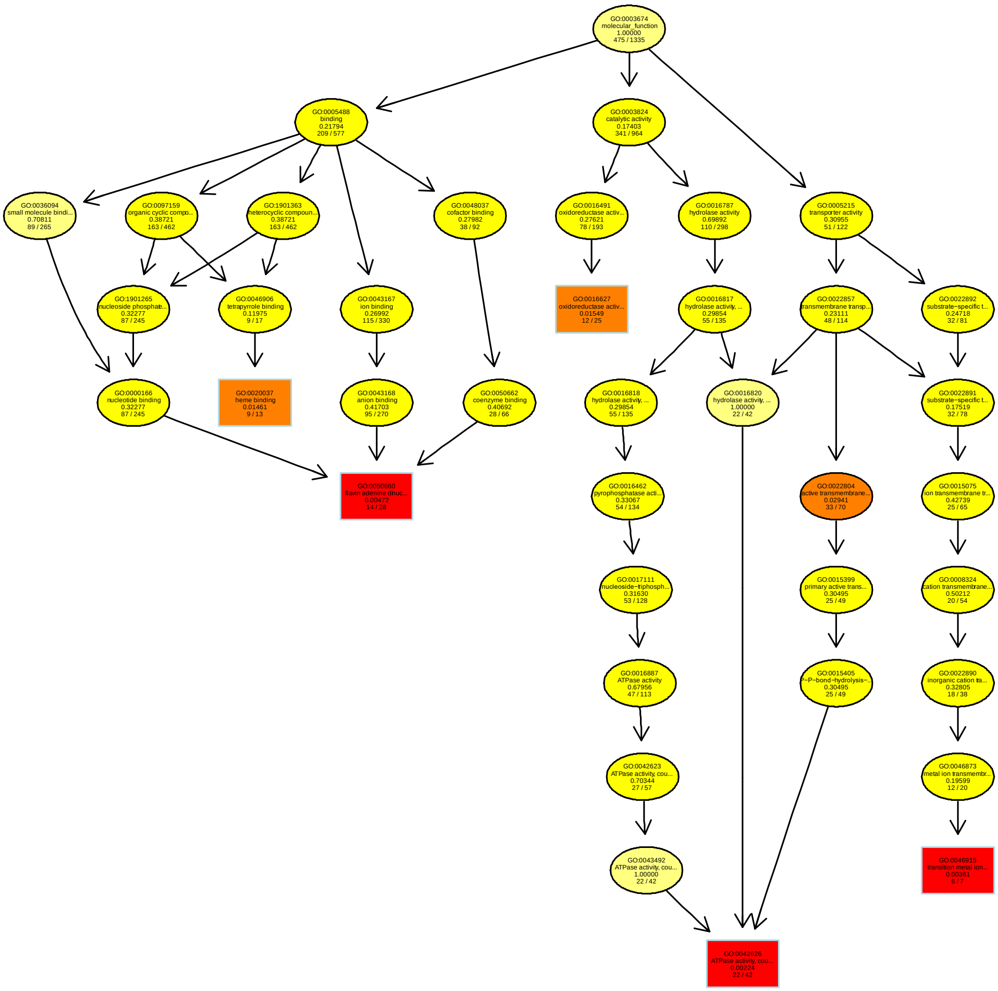

Figure S4. Directed Acyclic Graph of GO (MF) enrichment analysis.

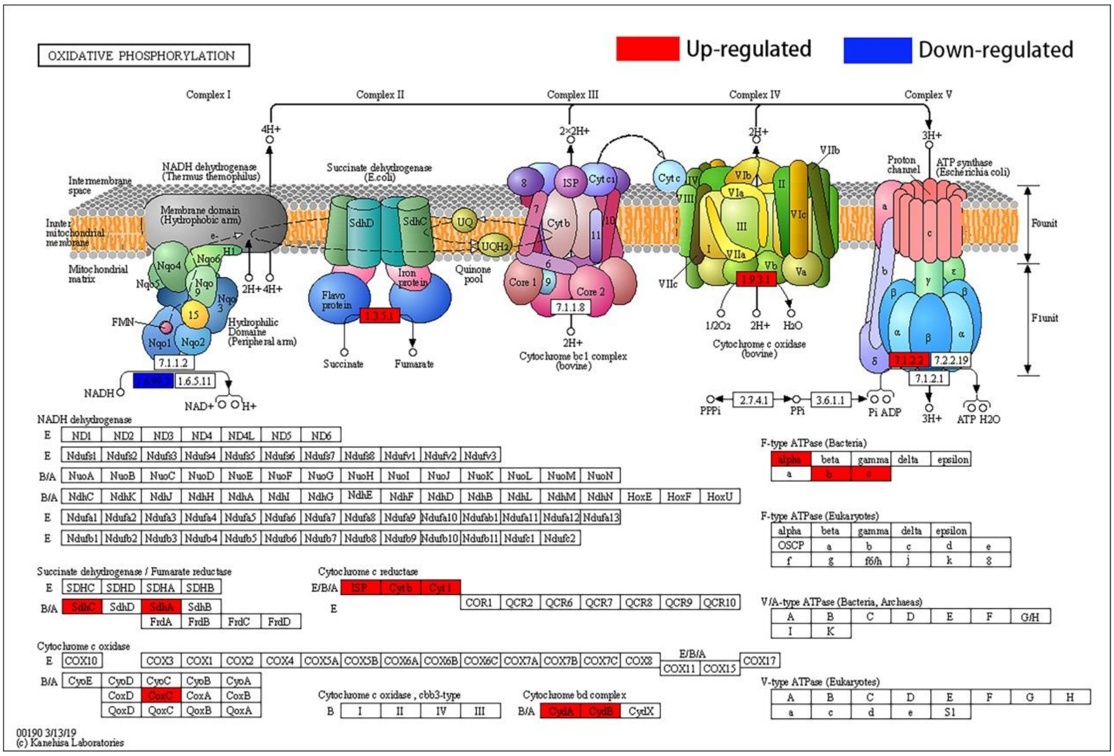

Figure S5. The KEGG pathways (ko00190) "Oxidative phosphorylation" mapped with DEGs.
